# Anterior Inferior Iliac Spine Avulsion Fracture: A Series of 5 Cases

**DOI:** 10.1097/MD.0000000000000562

**Published:** 2015-02-20

**Authors:** Sancar Serbest, Hacı Bayram Tosun, Uğur Tiftikçi, Birhan Oktas, Engin Kesgin

**Affiliations:** From the Faculty of Medicine (SS, UT, Birhan OKTAS), Department of Orthopaedics and Traumatology, Kırıkkale University, Kırıkkale; Faculty of Medicine (HBT), Department of Orthopaedics and Traumatology, Adıyaman University, Adıyaman; and Department of Orthopaedics and Traumatology (EK), Anamed Private Hospital, Mersin, Turkey.

## Abstract

Avulsion fractures of the pelvic apophyses rarely occur in adolescent athletes in the course of sudden strong contraction of muscle attached to growth cartilage. This injury may usually be misdiagnosed for tendon or muscle strain. Patient's history, physical examination, and radiologic studies are important for diagnosis. The literature includes only a few case reports but no case series as yet. The aim of this study was to present the results of 5 cases of anterior inferior iliac spine (AIIS) avulsion fractures treated conservatively.

The study included 5 patients (4 male, 1 female, mean age 13.6 years) who underwent conservative treatment for AIIS avulsion fractures and had an adequate follow-up. All patients were admitted to the emergency department and misdiagnosed as muscle strain. Three of them were football player, 1 skier, and 1 fighter.

Each patient was treated with immobilization and nonsteroidal anti-inflammatory drugs. At follow-up, all patients showed relief from their pain and mechanical symptoms and regained full range of motion and returned to their previous levels of activity.

Diagnosis requires careful attention to the physical examination and imaging. In this series, all pelvic avulsion fractures (100%) were managed successfully with a conservative approach. Good results and return to previous levels of activity can be achieved with conservative treatment.

## INTRODUCTION

Avulsion fracture of a pelvic apophysis in an adolescent athlete is a rare entity due to high stress of a violent forceful muscle contraction. Adolescent sport injuries are becoming a bigger problem with their increasing frequency and importance; 3% to 5% of all sport injuries are seen around groin region.^1^ These injuries are occurring mostly in competitive sports with hitting, running, and sudden direction changes in sprinting. Although some clues for diagnosis exist in patient's history, these injuries are still a big dilemma with their diagnosis and also treatment. Most of these injuries are misdiagnosed as strain and only medication was the treatment modality that causes delay in return to sports. Therefore, a carefully taken anamnesis and physical examination with radiographic confirmation are needed for exact diagnosis.^1–3^

## CASES

This is a retrospective case series study that included 5 patients (aged 12, 13, 14, 14, and 15, respectively) with anterior inferior iliac spine (AIIS) avulsion fractures who were treated conservatively. Written informed consent was obtained from the patients’ parents who participated in this case. Common complaints in all of the patients were sudden-onset groin pain that also caused limping. All patients were admitted to the emergency department and misdiagnosed for muscle strain. Three of them were football player, 1 skier, and 1 fighter. In all patients, AIIS avulsion fracture was detected after admission to orthopedic clinic because of continuing complaints.

**FIGURE 1:**
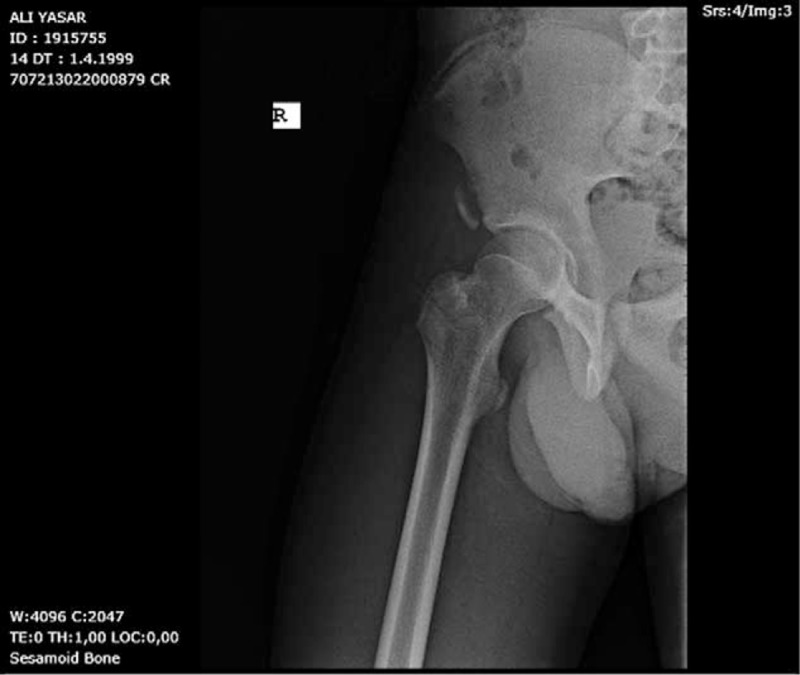
Pelvis anteroposterior x-ray shows a right anterior inferior iliac spine avulsion fracture in case 1.

**FIGURE 2:**
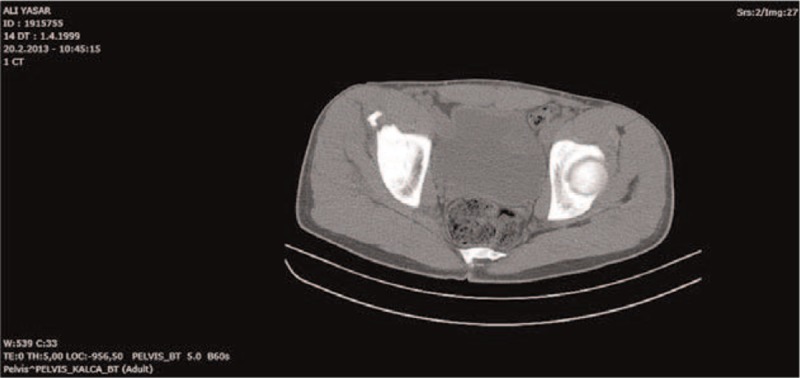
Axial computerized tomography scan of the pelvis showing the avulsion fracture of the left anterior superior iliac spine.

Case 1: Our first patient was a 14-year-old girl. She was injured during back fall while skiing. Mechanism of injury in the skier was uncontrolled hip/thigh motion that occurs when there is hyperextension of the hip joint and the flexion of the knee, as in the action of skiing. Her complaint was sudden-onset groin pain with disability to walk. She was immediately admitted to the emergency room. Only nonsteroidal anti-inflammatory drug was given which was ineffective for pain release. She has admitted to the orthopedic department after a while (Figures 1 and 2).

**FIGURE 3:**
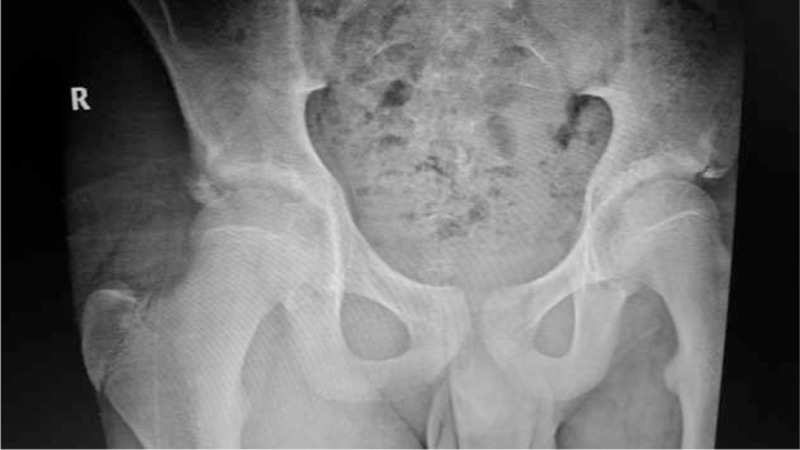
Pelvis anteroposterior x-ray shows a right anterior inferior iliac spine avulsion fracture in case 2.

**FIGURE 4:**
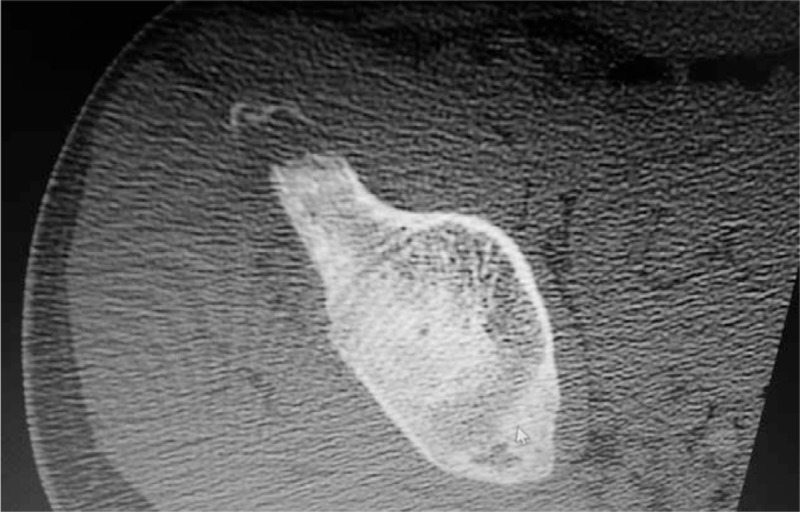
Axial computerized tomography scan of the pelvis showing the avulsion fracture of the left anterior superior iliac spine.

**FIGURE 5:**
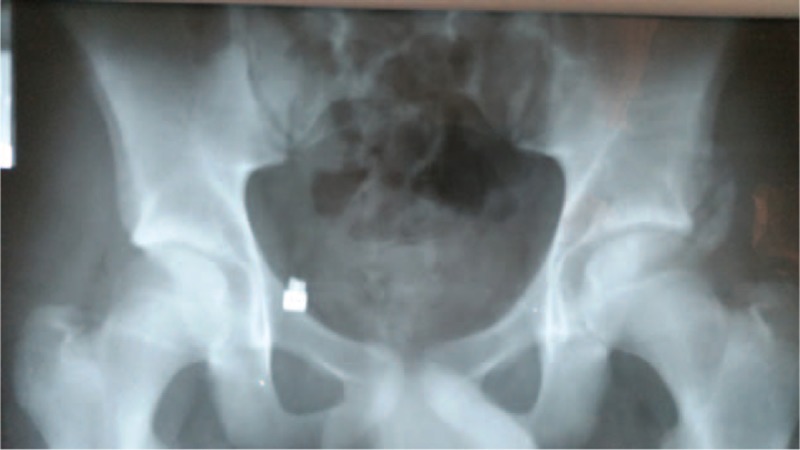
Pelvis anteroposterior x-ray shows a right anterior inferior iliac spine avulsion fracture in case 3.

**FIGURE 6:**
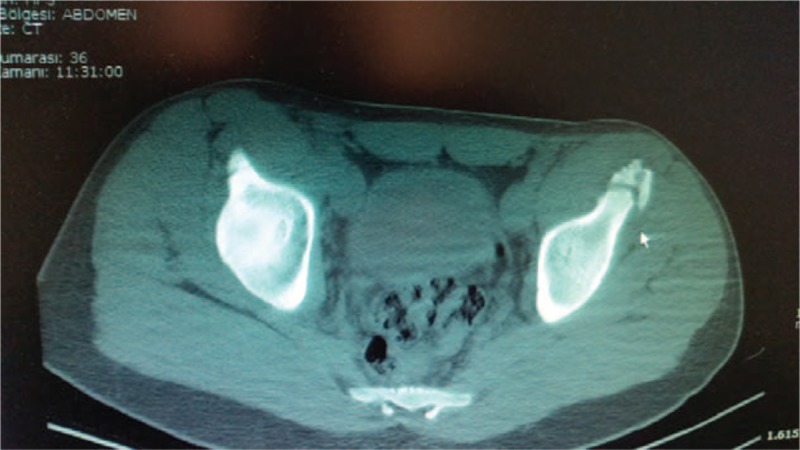
Axial computerized tomography scan of the pelvis showing the avulsion fracture of the left anterior superior iliac spine.

**FIGURE 7:**
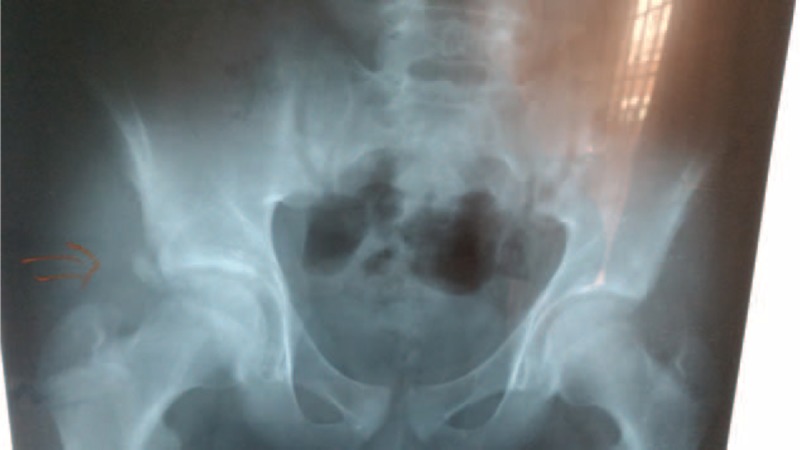
Pelvis anteroposterior x-ray shows a right anterior inferior iliac spine avulsion fracture in case 4.

**FIGURE 8:**
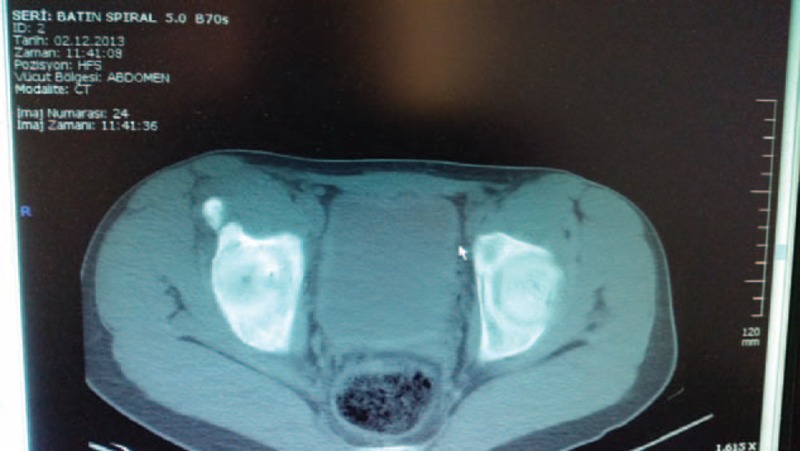
Axial computerized tomography scan of the pelvis showing the avulsion fracture of the left anterior superior iliac spine.

Cases 2, 3, and 4: These patients were male football players with ages 12, 14, and 15. They have begun to play without preconditioning and after a strengthful hit they all have had sudden-onset groin pain. Two of them on the same day and the other patient after 1 day have admitted to the emergency service. Because of the persistent pain even after medication usage, they were referred to the orthopedic department (Figures 3–8).

**FIGURE 9:**
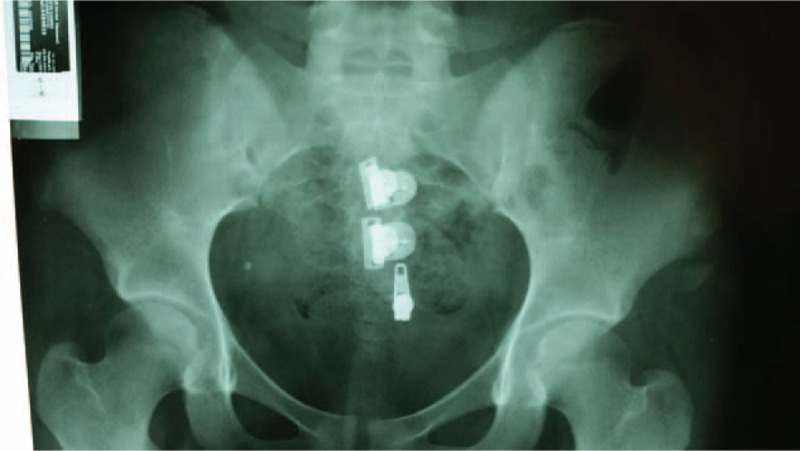
Pelvis anteroposterior x-ray shows a right anterior inferior iliac spine avulsion fracture in case 5.

**FIGURE 10:**
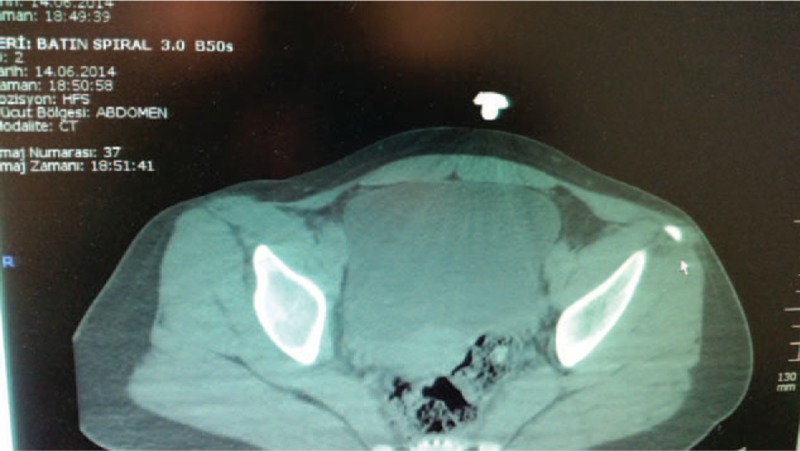
Axial computerized tomography scan of the pelvis showing the avulsion fracture of the left anterior superior iliac spine.

Case 5: A 13-year-old girl has admitted to the emergency service with a sudden-onset groin pain when she was hitting during fighting sport. She was also referred to our clinic with continuing pain (Figures 9 and 10).

Four of the patients were limping to right side whereas one to the left side. On physical examination, there was pain in palpation around the affected groin region and pain was spreading to anterior and anteromedial aspect of the thigh. Active flexion and passive extension was painful. On direct x-ray examination and computerized tomography (CT) scans, AIIS avulsion fractures were detected in all 5 patients.

In early period, all patients were treated with immobilization and nonsteroidal anti-inflammatory drugs. Conservative treatment consisted of bed rest, with the affected hip at 70° to 90° flexion for the first 2 weeks, symptomatic treatment of pain, and soft-tissue edema. Passive-assisted range-of-motion (ROM) exercises were initiated as soon as pain allowed. In the subacute phase, in order to obtain pain-free hip and knee ROM and improve quadriceps muscle function, we started rehabilitation that addressed ROM, strength, and neuromuscular control of the hip and knee. After 2 weeks of resting period, patients were mobilized with 2 crutches without weight bearing to the affected limb. After week 6, total weight bearing was allowed and at the end of week 8, running and basic conditioning was allowed. At the end of week 10, all sportive activities were allowed. All patients were able to compete and receive heavy training after week 10.

## DISCUSSION

AIIS avulsion fractures are more rare cases than other pelvic avulsion fractures. Its incidence is between 14.8% and 22.1%. More than 90% of these avulsion fractures are seen between 14 and 17 years of age mainly among boys.^4^ Because ossification of the pelvic ring continues until late adolescence, the weakest part of the musculotendinous unit is the apophysis with high incidence of avulsion injuries compared with muscles and tendons.^4,5^

AIIS avulsions are the results of sudden and forceful contraction of rectus femoris muscle concentrically or eccentrically. While concentric contraction occurs on the acceleration phase of the push-off, eccentric contraction is the end result of deceleration. The most common injury mechanism is concentric contraction during the start of sprinting although eccentric contraction is also seen after sliding-type injuries.^6^ During the injury, although the hip joint is hyperextended and the knee joint is in flexed position, the muscle rectus femoris is more prone to this type of injury while hitting the ball in a football play.^7^ The most common activities causing avulsion fractures are sprint running, steeplechase, sudden direction-changing sportive activities, sudden accelerating and decelerating activities, and uncontrolled football hitting. Less-common mechanism is excessive passive elongation of the musculotendinous unit during gymnastic movements.^1,5^ One of our case was injured during back fall while skiing, by hitting the ball during football match in other 3 cases, and during hitting in fighting sport in another case.

During apophyseal avulsion of pelvis, loud sound of rupture is heard which is followed by sudden severe pain with disability to move the limb.^8^ The muscles around the injured region cause severe pain while active contraction. Although some local tenderness exists on palpation, deep palpation is needed to localize an avulsion of AIIS.^8,9^ Practitioners interpret these injuries mostly as strain. All of our patients are also misdiagnosed as simple muscle strain in first admission to emergency service. Avulsed fragment could be seen on direct x-rays. These must also be differentiated from os acetabuli (an accessory ossicle at the superior rim of the acetabulum), traumatic myositis ossificans of the rectus femoris, and bone tumors. CT can be used in order to determine exact displacement of the fragment. If the fracture displacement is more than two centimeters, surgically open reduction and internal fixation are needed.^2,10^ They sometimes resemble to Salter–Harris type 1 or 2 epiphyseal injuries. Direct comparative x-rays with contralateral side are very useful in exact diagnosis.^1,8^

The treatment of AIIS avulsion fractures is mostly conservative. Only limited cases exist in the literature that are treated with open reduction and internal fixation. They also have similar results with conservatively treated cases. Surgery is only indicated in cases with displacement more than two centimeters, nonunions, and early recovery-needed professional athletes.^10^ Fibrous unions with long duration of persistent pain are also evaluated for surgical excision depending on displacement of fragment. In conservative treatment, bed rest, analgesic medication, and 5-staged rehabilitation program are included.^7,9^

Total recovery achieved between third week and fourth month. Early return to sportive activities may result in displacement and prolonged union. Although the fragment is displaced more distally than its original insertion due to the contraction of the rectus femoris muscle, it has not been shown any limitation affecting their sportive activities. Healing is achieved generally with callus formation. The functional results of conservative and surgical treatments are similar in returning to preinjured athletic level. Also, in all of our cases, radiological healing with callus formation and returning to preinjured ROM is achieved at the end of week 10.^8^

As a result, AIIS avulsion fractures are seen very rarely in adolescent athletes with sudden or uncontrolled forceful contraction of rectus femoris muscle. These injuries are very convenient to be misdiagnosed as a simple strain and late diagnosis may cause chronic pain with decreased sportive performance in the future. Therefore, comparative anterior–posterior pelvic x-rays are needed not to miss avulsions in adolescents; also in some instances, more advanced scanning methods must be considered.
